# The ubiquitin system targets translocated EspH to proteasomal degradation

**DOI:** 10.1080/19490976.2025.2595775

**Published:** 2025-12-04

**Authors:** Ipsita Nandi, Efrat Zlotkin-Rivkin, Hanan Schoffman, Benjamin Aroeti

**Affiliations:** a Department of Biological Chemistry; The Alexander Silberman Institute of Life Sciences; The Hebrew University of Jerusalem, The Edmond J. Safra Campus – Givat Ram, Jerusalem, Israel; b Department of Cell and Developmental Biology; The Alexander Silberman Institute of Life Sciences; The Hebrew University of Jerusalem, The Edmond J. Safra Campus – Givat Ram, Jerusalem, Israel; c Proteomics and Mass Spectrometry Unit, The Alexander Silberman Institute of Life Sciences; The Hebrew University of Jerusalem, Jerusalem, Israel

**Keywords:** Enteropathogenic *E. coli* (EPEC), type III secreted effectors, EspH, effector polyubiquitylation, proteasomal degradation, effector post-translational modifications, mass spectrometry, host-pathogen interactions

## Abstract

EspH is an effector protein secreted by the type III secretion system of various pathogenic *Escherichia coli* strains, including enteropathogenic *E. coli* (EPEC). The ability of EspH to inhibit host RhoGTPases, disrupt the actin cytoskeleton, and induce host cell cytotoxicity has been well-documented. Mass spectrometry analysis of EspH translocated into EPEC-infected cells revealed that a lysine at position 106 (K106) is modified with ubiquitin. Immunoblotting using the FK2 anti-ubiquitin antibodies has confirmed these results, suggesting that EspH undergoes polyubiquitylation. Prediction algorithms have identified a single ubiquitylation site at K106 and a phosphodegron in EspH. Moreover, we show that wild-type (EspH_*wt*_), but not the EspH_*K106R*_ mutant, is subjected to degradation following translocation in an MG132-sensitive manner, indicating that the proteasome degrades the polyubiquitylated effector following translocation. Finally, we show that translocated EspH_*K106*R_ induces higher cytotoxicity than translocated EspH_*wt*_. EspH_*wt*_ translocated into MG132-pretreated cells also displayed higher cytotoxicity levels than EspH_*wt*_ in untreated cells. These data reinforce the idea that EspH is polyubiquitylated and that the host proteasome degrades the translocated effector, possibly limiting its ability to toxicate the host cells. Additional implications of these effects on bacterial-host interactions are discussed.

## Introduction

Post-translational modifications (PTMs) can control proteins’ activity and lifetime (stability).^1^ A primary PTM involved in regulating protein stability is ubiquitylation, which functions through the ubiquitin-proteasomal system (UPS).^2^ The UPS controls broad cellular pathways, including signaling cascades, cell growth, differentiation, and proliferation in health and disease.^3^^,^^4^ The ubiquitin system has also been implicated in modulating bacterial host-pathogen interactions.^5‐10^

Although pathogenic bacteria and viruses lack a functional ubiquitin system, many have evolved sophisticated strategies to control it in their eukaryotic hosts. One of the most exciting ways pathogenic bacteria interact with the eukaryotic ubiquitin pathway is through secreted effector proteins. Strategies employed by bacterial effectors to hijack and manipulate the ubiquitin signaling pathways are diverse.^5^^,^^6^^,^^8‐13^ For example, specific bacterial protein effectors can mimic the activity of components of the ubiquitin-proteasome system, e.g., the E2 and E3 ubiquitin ligases, and ubiquitinate host proteins. Others can bind and inhibit E2 or E3 ubiquitin ligases or act as deubiquitinases that remove ubiquitin from host proteins.^6^^,^^14^^,^^15^ Given the numerous processes the ubiquitin system controls in eukaryotic cells, including roles in adaptive and host innate defense immunity,^14^^,^^16^^,^^17^ it is not surprising that the targeting of the eukaryotic ubiquitin system would give an evolutionary advantage to the pathogenic bacteria,^5‐7,9,10^and that the ubiquitin system serves as an attractive target for the development of therapeutics against bacterial pathogens.^18^^,^^19^

Enteropathogenic *E. coli* (EPEC) and enterohemorrhagic *E. coli* (EHEC) are diarrheagenic human bacterial pathogens that cause acute gastroenteritis and are therefore targets for intensive vaccine and therapeutic development.^20‐22^ These enteric pathogens, along with the rodent pathogen *Citrobacter rodentium*, belong to a family called the attaching and effacing (A/E) pathogens, which form a unique histological lesion at bacterial attachment sites in the intestinal epithelium. The bacteria’s virulence largely depends upon their capacity to construct a nanoinjection machinery called the type III secretion system (T3SS), which mediates the injection (translocation) of effector proteins from the bacterial cytoplasm into the host cells.^23^

Here, we report a case in which a toxic type III secreted effector, EspH, expressed by EPEC (and other A/E pathogens) may undergo PTMs, including polyubiquitylation at a specific lysine (K106), and degradation by the UPS. EspH has been reported to disrupt the host cell actin cytoskeleton,^24^^,^^25^and desmosomes,^26^inhibit bacterial phagocytosis (invasion)^27^ and Erk signaling,^28^and induce host cell cytotoxicity. At the molecular level, many of these functions have been attributed to the ability of EspH to inhibit the host cell Rho,^26^^,^^27^^,^^29^^,^^30^and Rab^31^ GTPases. The effects of EspH polyubiquitylation on its lifetime and host cell cytotoxicity have been studied herein and discussed in the context of host-pathogen interactions.

## Materials and methods

### Bacterial strains, antibodies, plasmids, and primers

Bacterial strains, antibodies, and reagents used in this study are listed in Table S1.

### Cell culture

HeLa and CaCo−2_BBe_ cells (semi-polarized) (Table S1) were cultured, as previously described.^30^

### Bacterial pre-activation (priming) and cell infection

Before infection, the T3SS of bacterial strains was activated in plain high-glucose Dulbecco’s Modified Eagle Medium (DMEM) for 3  hrs in a CO_2_ incubator (37 °C, 5% CO_2_, 95% humidity), as described.^30^ EPEC-Δ*esp*H was transformed with a pSA10 vector encoding EspH_*wt*_-6XHis (6XHis-tag)- Stretavidin binding peptide (SBP-tag) or EspH_*K106R*_-6XHis-SBP to generate the EPEC-Δ*esp*H/pEspH_*wt*_ and EPEC-Δ*esp*H/pEspH_*K106R*_ complemented strains. EspH expression was induced by supplementing the activation medium with isopropyl-*β*-D-thiogalactopyranoside (IPTG; 0.05 mM) during the last 30  mins of activation. Cell infection was performed using pre-activated infection medium in a CO_2_ incubator at 37  °C for the indicated times. Bacterial infection was performed at a multiplicity of infection (MOI) of ~100.

### Pulldown (precipitation) of translocated EspH for mass spectrometry (MS) analysis

HeLa cells, grown to 70% confluency in a Nunclon Delta Treated Square BioAssay Dish (ThermoFisher Scientific, 166508), were washed twice with warm (37 °C) DMEM, treated with the proteasome inhibitor MG132 (10 µM) for 3 hrs at 37 °C, washed twice with plain DMEM, and then infected for 90  mins at 37  °C with pre-activated bacteria. Effector protein expression was induced with IPTG. Cells were then washed three times with ice-cold PBS and lysed in ice-cold lysis buffer [50  mM Tris (pH 7.4), 150  mM NaCl, 1% NP−40, 1  mM EDTA, 0.25% Sodium deoxycholate, 10  mM *N*-Ethylmaleimide (NEM) (Sigma, E3876)] supplemented with protease and phosphatase inhibitors (Sigma, PPC1010−1 ML). The lysate was gently homogenized 10 times through a 1-ml pipette tip and then 10 times through a 19-G needle. Following a 30-minute incubation at 4 °C, the lysate was centrifuged (5,000 g, 10 mins, 4 °C). The cell lysate was then incubated with 60 µL of Streptavidin (StAv) Agarose beads (Sigma, S1638, 50% slurry pre-washed with lysis buffer) for 4 hrs at 4 °C with end-to-end rotation. Beads were then washed three times by centrifugation (300 g, 2 mins, 4 °C) with lysis buffer and three times with Tris-HCl buffer [50 mM Tris (pH 7.4), 150 mM NaCl], dried using a Hamilton syringe, and subjected to analysis by Liquid Chromatography-Mass Spectrometry (LC-MS). Each experiment was performed in a quadruple.

### Identification of potential PTMs in translocated EspH by MetaMorpheus

Pulled-down EspH was analysed by LC-MS, and PTMs were identified using MetaMorpheus database search software, version 1.0.5.^32^ A search was performed using the calibration module, followed by the PTM discovery module using all “common biological” and “common artifact” PTMs checked and the addition of variable modification, “GG (Ubiquitylation Site) on K” and in the case of the K106R strain, a custom modification of GG on R. Following the PTM discovery the search module was used. Both human and EPEC O127:H6 (strain E2348/69) reference proteomes from Uniprot were used in the search. The combined search database contained 109344 non-decoy protein entries, including 296 contaminant sequences.

### LC-MS analysis

Analysis was performed at the Stein Family mass spectrometry center in the Silberman Institute of Life Sciences, Hebrew University of Jerusalem. Protein samples were prepared and analyzed as previously described.^30^ In brief: Proteins were denatured on beads using 8 M urea/10 mM DTT/25 mM Tris-HCl (pH 8), for 30 mins at room temperature, followed by alkylation with 55 mM iodoacetamide at room temperature in the dark. Urea was diluted to < 1 M by adding eight volumes of Tris-HCl. Digestion was performed using sequencing-grade trypsin (Promega) at 0.4 µg per sample overnight at 37 °C with gentle shaking. The resulting peptides were desalted using homemade C18 stage tips. 0.35 µg peptides (measured by OD280) were used for MS analysis. MS analysis was performed using a Q Exactive-HF mass spectrometer (Thermo Fisher Scientific, Waltham, MA, USA) coupled online to an Ultimate 3000 Dionex UHPLC (Thermo Fisher Scientific, Waltham, MA, USA). Peptides were separated on a 73-minute acetonitrile non-linear gradient run at a flow rate of 0.30 μl/min on a reverse-phase 25-cm-long C18 column (Aurora Ultimate XT 25 × 75, IonOpticks, AU). Survey scans (300–1,650 m/z, target value 3E6 charges, maximum ion injection time 20 ms) were acquired and followed by higher energy collisional dissociation (HCD) based fragmentation (normalized collision energy 27). A resolution of 60,000 was used for survey scans, and up to 15 dynamically chosen, most abundant precursor ions with a “peptide preferable” profile were fragmented (isolation window 1.6 m/z). The MS/MS scans were acquired at a resolution of 15,000 (target value: 1E5 charges; maximum ion injection time: 25 ms). Dynamic exclusion was 20 sec. Data were acquired using Xcalibur software (Thermo Scientific). To avoid carryover, the column was washed with 80% acetonitrile and 0.1% formic acid for 25 min between samples.

### Sodium dodecyl sulfate polyacrylamide gel electrophoresis (SDS-PAGE) and immunoblotting (IB)

SDS-PAGE and IB were performed as described.^33^ Band intensities of the immunoblots were quantified in Fiji (National Institutes of Health [NIH]) with background subtraction.

### Pulldown of translocated EspH and analysis of its ubiquitylation by IB

EspH pulldown after cell infection was performed as previously described,^30^ except that the HeLa cells were cultured on two 15-cm plates (**80%** confluence) and treated with MG132 (10 µM) for 3 hrs at 37 °C before infection. Pulled-down EspH and polyubiquitylated EspH were detected following SDS-PAGE and IB using anti-SBP and anti-mono and polyubiquitin (FK2) antibodies, respectively.

### Analysis of EspH degradation

HeLa (3 × 10^5^ cells/well) or Caco−2_BBe_ (5 × 10^5^ cells/well) cells were cultured in 6-well plates and incubated in a CO_2_ incubator for 48 hrs until reaching ~80% confluence (HeLa) or a complete cell monolayer (Caco−2_BBe_). For each time interval, cells cultured in three wells of a 6-well plate were treated with MG132 (10 µM) or an equivalent amount of DMSO (control) for 3 hrs and then infected with pre-activated EPEC expressing EspH*_wt_* or EspH_*K106R*_ for 30 mins (Figure 2A, indicated with green arrow). Infection was terminated by replacing the medium, bathing the cells with DMEM containing 200 μg/ml Gentamycin. The zero time point is designated as the time at which the cells were lysed immediately after adding Gentamycin (Figure 2A, red arrow). Cells were then further incubated in the CO_2_ incubator at 37 °C for the indicated time intervals. At each time point, cells were washed three times with ice-cold PBS and lysed with an NP40-based lysis buffer. Lysates from three wells were then combined, and EspH was precipitated (*P*) using StAv agarose beads, as described.^30^ Precipitated EspH was detected following SDS-PAGE and IB using anti-SBP antibodies. Approximately 30 µg (Bradford Reagent; Sigma-Aldrich B6916) of lysate protein was analyzed by SDS-PAGE and IB, and anti-*α*-tubulin antibodies were used to evaluate the lysate protein levels.

### Host cell cytotoxicity measurements

#### Propidium Iodide (PI) uptake

HeLa cells seeded in a black clear-bottom 96-well plate (2 × 10^4^ cells/well) were incubated for 48 hrs in a CO_2_ incubator, and the PI-uptake assay to evaluate lytic host cell death was performed, as described ^34^ with some modifications. Cells were treated with MG132 (10 µM) or an equivalent amount of DMSO for 3 hrs, then infected with pre-activated bacteria. The cell medium was then replaced with PI (5 µg/ml)/IPTG (0.05 mM)-containing primed bacteria, and the cells were incubated in a CO_2_ incubator for 30 mins (Figure 3A, green arrow). Then, the medium bathing the cells was replaced with Gentamycin (200 μg/ml) containing medium for the indicated time points (Figure 3A, red arrow). The PI fluorescence was measured (BioTek Synergy H1 plate reader; 520-nm excitation and 620-nm emission wavelengths) every 30 mins for up to 300 mins.

**Figure 1.**
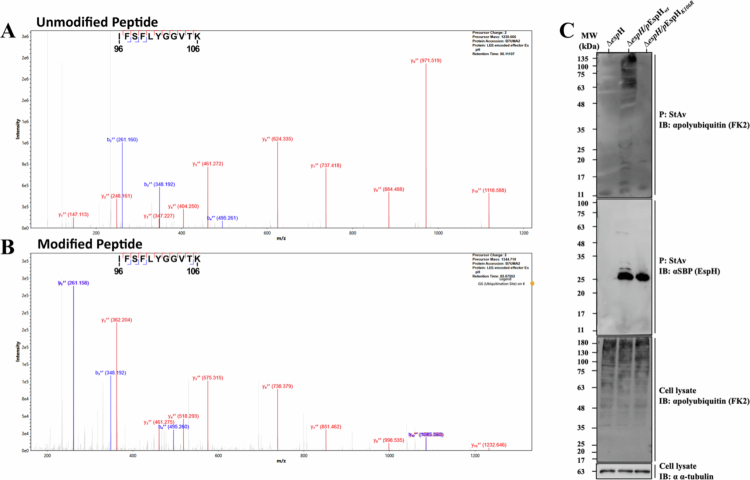
Representative MS/MS spectra of the IFSFLYGGVTK peptide of EspH. A. Unmodified peptide. Representative MS/MS spectrum of the IFSFLYGGVTK peptide corresponding to positions 96 to 106 in the EspH protein. The b ions (blue) and y ions (red) cover the entire peptide, enabling accurate sequence and modification identification. Fragment ion masses are shown in the graph, precursor mass 1230.666 Da. B. Modified peptide. Representative MS/MS spectrum of the IFSFLYGGVTK peptide with ubiquitylation site on K106. Precursor mass of the parent ion is 1344.710, which represents a mass shift of 114.044 Da from the unmodified sequence, corresponding to the monoisotopic mass of a Gly-Gly dipeptide. The mass difference between the y1 ions in panels 1a and 1b corresponds to a localization of the Gly-Gly on lysine in position 106. C. Translocated EspH*_wt_*, but not EspH*_K106R_*, is modified with polyubiquitin identified by IB. HeLa cells were infected with the indicated EPEC strains, lysed, and subjected to precipitation experiments, as described in Materials and Methods. SBP-tagged EspH*_wt_* or EspH*_K106R_* was precipitated (*P*) with streptavidin (StAv) beads. Anti-SBP and anti (*α*) -polyubiquitin (FK2) antibodies were used to identify the endogenous polyubiquitin chains (**upper**) and the precipitated EspH (**middle**) by IB. Cell lysates probed with FK2 and anti-α-tubulin antibodies are shown (**lower**). Representative gels from 3 independent experiments are shown.

#### **LDH release**

HeLa cells (2 × 10^4^ cells/well) and Caco−2_BBe_ cells (3 × 10^4^ cells/well) were seeded in a flat-bottom 96-well plate and incubated in a CO_2_ incubator for 48 hrs until reaching ~80% (HeLa) or complete (Caco−2_BBe_) cell confluence was reached. The LDH assay was applied as described^31^ with some modifications. Bacteria were primed in plain DMEM for 3 hrs. IPTG (0.05 mM) was added to the activated bacteria medium after 2.5 hrs of incubation. The cell medium was then replaced with IPTG-activated bacteria, and the cells were incubated in a CO_2_ incubator for 30 mins (Figure 3B, green arrow). Then, the medium bathing the cells was replaced with Gentamycin (200 μg/ml) containing medium for 2 hrs (Figure 3B, red arrow). For MG132 treatment, cells were incubated with 10 µM MG132 for 3 hrs before infection. Cells pretreated with the equivalent amount of DMSO served as controls in these experiments. The LDH release assay was performed on the cell culture medium according to the protocol provided in the CytoTox 96® Non-Radioactive Cytotoxicity Assay kit (Promega # G1780).

### Statistical analysis

The GraphPad Prism v. 8.4.3 software was used for statistical analysis and graphing. A one-way ANOVA followed by Bonferroni’s multiple-comparison test was applied to determine the statistical significance. The significance is indicated by asterisks, as follows: ****p ≤ 0.0005; ***p > 0.0005; **p < 0.005; *p > 0.005; ns, non-significant *p* > 0.05. A *p*-value < 0.05 indicates a statistically significant difference.

## Results

### Mass-spectrometry analysis reveals post-translational modifications, including ubiquitylation at K106, in translocated EspH

MS analysis of translocated EspH following cell infection with EPEC-Δ*esp*H/pEspH_*wt*_^30^ by the MetaMorpheus software^32^ revealed several PTMs, including a notable ubiquitylation on lysine K106 (Table S2 and Figure 1A and 1B). Ubiquitylation at K106 was not observed in the translocated EspH_*K106R*_ mutant (Table S2). Several other PTMs were identified in this way, including methylation at K75 and several hydroxylations (Table S2). However, in the present study, we focused on analyzing the ubiquitylation of EspH, which was consistently detected across all replicates at 0.55% (STDEV 0.07), as estimated from peptide intensities. HeLa cells were infected with EPEC-Δ*espH*, EPEC-Δ*espH*/pEspH_*wt*_, or EPEC-Δ*espH*/pEspH_*K106R*_, lysed, and EspH was pulled down with Streptavidin (StAv) beads. The pulled-down proteins were analyzed by SDS-PAGE followed by IB using anti-ubiquitin (mono- and polyubiquitin) FK2 antibodies, as described in Materials and Methods and Table S1. The results show a dramatic increase in the appearance of multiple protein bands at the high molecular weight range when EspH was pulled down from cells infected with EPEC-Δ*espH*/pEspH_*wt*_, but not from cells infected with EPEC-Δ*esp*H, or cells infected with EPEC-Δ*esp*H/pEspH_*K106R*_ (Figure 1C, upper). As expected, probing with anti-SBP did not show an EspH protein band in EPEC-Δ*espH*-infected cells. However, the anti-SBP antibodies unravel a few relatively weak protein bands at an apparent molecular weight higher than the native ~25kDa EspH-SBP protein band only in EPEC-Δ*espH*/pEspH_*wt*_ infected cells (Figure 1C, middle)**,** suggesting that they may have limited capacity to recognize ubiquitylated or otherwise modified EspH proteins. As expected, high-molecular-weight protein bands, likely representing polyubiquitylated proteins, were observed in all cell lysates probed with the FK2 antibodies (Figure 1C, lower).

Finally, in this context, the Rapid scale UBIquitination detection (RUBI) prediction algorithm [^35^ http://old.protein.bio.unipd.it/rubi/] has also predicted the ubiquitylation of EspH at K106 (Figuer S1). Moreover, the amino acid sequence of EspH was analyzed using the Degronopedia prediction analysis platform to identify potential degron motifs [^36^https://degronopedia.com/]. Upon further assessment, it was observed that the three highest-scoring degrons are located towards the C-terminus of the K106 (not shown). One of these top three degrons has been identified as a phosphodegron (Figure S1). A phosphodegron is a phosphorylated residue(s) on a protein that interacts with an interaction domain in an E3 ubiquitin ligase, facilitating the substrate’s linkage to the ubiquitin conjugation machinery.^37^ Identifying these putative degrons, particularly the phosphodegron, provides additional compelling evidence for the polyubiquitylation of EspH and will be the subject of further investigation. These data, combined, firmly suggest that EspH undergoes polyubiquitylation at K106 upon translocation, targeting the protein for proteasomal degradation.

### Translocated EspH is subjected to ubiquitin-dependent proteasomal degradation

**Figure 2.**
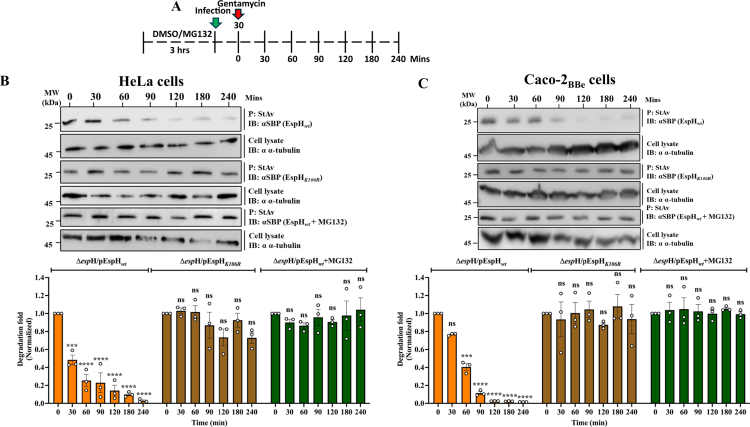
EspH*_wt_*, but not EspH*_K106R_,* is subjected to proteasomal degradation: (A) Schematic description of the course of the experiment. The degradation assay was performed in HeLa (B) and Caco-2_BBe_ (C) cells, as described in Materials and Methods. Representative gels from 3 independent experiments are shown. Quantitative analysis of the EspH band levels included normalization of the EspH band intensity to that of tubulin, and the resulting values were further normalized to those measured at time zero. Results are mean ± SE.

Next, we aimed to elucidate whether EspH’s polyubiquitylation at K106 is involved in proteasomal degradation of the effector protein. As described in Materials and Methods and Figure 2A, HeLa cells were infected with EPEC-Δ*espH*/pEspH_*wt*_ or EPEC-Δ*espH*/pEspH_*K106R*_. Cells were lysed, and EspH was pulled down by StAv-beads and analyzed by SDS-PAGE and IB. Data in Figure 2B show a time-dependent diminishment of EspH_*wt*_ but not the EspH_*K106R*_ protein band levels, suggesting that EspH is indeed subjected to proteasomal degradation and that the polyubiquitylated K106 residue plays a role in the process. Next, we investigated whether EspH undergoes proteasome-dependent degradation. HeLa cells were pretreated with the proteasomal inhibitor MG132, infected with EPEC-Δ*espH*/pEspH_*wt*_, lysed, and EspH was detected by IB. Data showed no effect on EspH levels as a function of infection time (Figure 2B). These results suggest that EspH*_wt_* undergoes proteasomal-dependent degradation after translocation. Similar results were observed in Caco−2_BBe_ cells (Figure 2C), suggesting that proteasomal degradation of EspH is not cell-type specific and can occur in epithelial cells that are more physiologically relevant for EPEC infection, such as the Caco−2_BBe_ cells.^38^

### EspH-induced host cell cytotoxicity is increased upon infection with EPEC-EspH*_K106R_* or in response to proteasome inhibition

**Figure 3.**
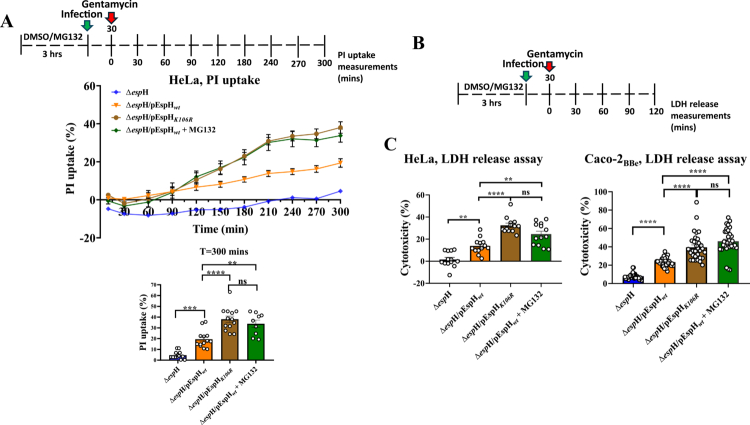
Effects of EspH*_wt_*, EspH*_K106R_,* and EspH*_wt_* translocated into pretreated or MG132-treated cells on cell cytotoxicity. A schematic description of the experimental procedure is shown in the upper panels. HeLa cells (A and B) or Caco-2_BBe_ cells (C) were infected with the indicated EPEC strains, and the effect on host cell cytotoxicity was measured using the PI-uptake (A) or LDH release (B and C) assays, as described in Materials and Methods. Results are the mean ± SE of 9 independent measurements. Error bars are not visible when smaller than the data point symbol.

To study the effects of EspH polyubiquitylation on host cytotoxicity, HeLa (Figure 3A,B) or Caco−2_BBe_ (Figure 3B) cells were infected with EPEC-Δ*espH*, EPEC-Δ*espH*/pEspH_*wt*_, or EPEC-Δ*espH*/pEspH_*K106R*_, under conditions described schematically in the upper panels of plots A and B, and in Materials and Methods. The effects on cell cytotoxicity were assessed by the PI-uptake assay in a time-dependent manner (Figure 3A) or by the LDH-release assay (Figure 3B), as described in Materials and Methods. Consistent with previous observations,^30^^,^^31^ cell infection with EPEC-Δ*espH*/pEspH_*wt*_ resulted in higher time-dependent PI uptake and LDH release levels than EPEC-Δ*espH*-infected cells. The results also show higher PI uptake and LDH-release levels in cells infected with the ubiquitylation-deficient EspH_*K106R*_ mutant compared to the wild-type effector, suggesting that EspH polyubiquitylation and subsequent effector degradation play a role in determining the levels of EspH-induced host cell cytotoxicity. This conclusion is further supported by data showing increased PI uptake and LDH release levels in cells treated with MG132 and infected with EPEC-Δ*espH*/pEspH_*wt*_.

## Discussion

Our results suggest that the Rho GTPase modulating EPEC effector, EspH, undergoes polyubiquitylation at lysine 106 and proteasomal degradation (Figures 1 and 2). Our study is the first to report that an A/E pathogen effector protein is subject to this process. These findings seem relevant to EspH’s activities because inhibiting ubiquitylation by mutating K106 to Arg or by treating with the proteasomal inhibitor MG132 increased the EspH effect (Figure 3). Finally, the finding of a predicted phosphodegron motif (Figure S1) is also consistent with the hypothesis that translocated EspH is subjected to regulated degradation by the host UPS. Notably, however, because of the limited levels of native (endogenous) EspH molecules injected into the host by EPEC*_wt_* ^39^, and the lack of means to detect it, our experiments were restricted to the epitope-tagged version of the effector protein overexpressed in bacteria; hence, the relevance of our findings to the native effector protein awaits future exploration.

What is the functional significance of EspH proteasomal degradation within the host? A fundamental effect of EspH is the induction of host cell cytotoxicity and the inhibition of bacterial invasion into the host cell, both of which are attributed to its ability to inhibit host cell Rho GTPases.^24^^,^^27^^,^^29^^,^^30^ EspH may antagonize the activity of another EPEC effector, Map, which acts as a Rho guanine nucleotide exchange factor [GEF^29^], i.e., as a Rho GTPase activator. We postulate that regulated degradation of translocated EspH is vital for maintaining defined effector levels within the host cells, enabling adequate host cell viability and optimal bacterial colonization.

In this context, EspH may be similar to other bacterial effectors. One example is the *Salmonella* type III secreted effectors, SopE and SptP. SopE acts as a GEF that activates signaling by two Rho GTPase family members, Rac−1 and Cdc42, thereby promoting the actin cytoskeletal reorganization needed for bacterial invasion into host cells. In contrast, the SptP effector acts as a GTPase-activating protein (GAP) that deactivates Rac and Cdc42, allowing the recovery of the actin cytoskeleton. For successful colonization, it has been suggested that the activity of these two effectors must be temporally regulated within the host cell. The mechanism enabling such coordinated activity has been attributed to differential degradation of effector proteins by the host UPS. SopE and SptP are delivered in equal amounts during infection. However, SopE undergoes polyubiquitylation and rapid proteasome-dependent degradation following translocation, while SptP is degraded much more slowly.^40^

Another example is *Yersinia pseudotuberculosis*, an extracellular bacterial pathogen that injects several effectors that interfere with the actin cytoskeleton dynamics, thereby blocking its invasion. The *Y. pseudotuberculosis* type III secreted YopE effector contributes to virulence by depolymerizing the host cell actin cytoskeleton by inhibiting Rho GTPases. This activity also prevents the formation of pores in host membranes and subsequent cell death, thereby prolonging colonization of the host.^41‐45^ YopE is polyubiquitylated on lysine 75 and targeted for proteasome degradation.^46^ Although the physiological significance of this process is not entirely clear, studies have indicated that it may help the host to sense the bacteria and repress bacterial anti-host cell activities.^46^^,^^47^

Why are Rho GTPase modulating effectors programmed to be degraded? Seminal studies by the Shao laboratory could provide an intriguing explanation. These studies have shown that a battery of bacterial toxins and pathogenic bacterial type VI secretion system components can inactivate Rho GTPases and activate the pyrin inflammasome, which is involved in the innate immune response against pathogens.^48^^,^^49^ Hence, eliminating the Rho GTPase modulating effectors by the UPS may represent an efficient way for bacterial pathogens to evade the pyrin-dependent innate immune response, extending their survival in the host.

## Supplementary Material

Supplementary Material Supplementary Material.

Supplementary Material Supplementary Material.

Supplementary Material Table S1: Cells, Bacterial Strains, Antibodies, Reagents/Kits, Materials, and Software.

## Data Availability

The data supporting this study’s findings are available from the corresponding author, BA, upon reasonable request.
