# Supplementary figures and images for: The ubiquitin system targets translocated EspH to proteasomal degradation
Source: Gut Microbes. 2025 Dec 4;17(1):2595775. doi: 10.1080/19490976.2025.2595775 (PMC12688225; doi:10.1080/19490976.2025.2595775)

Fig. S1

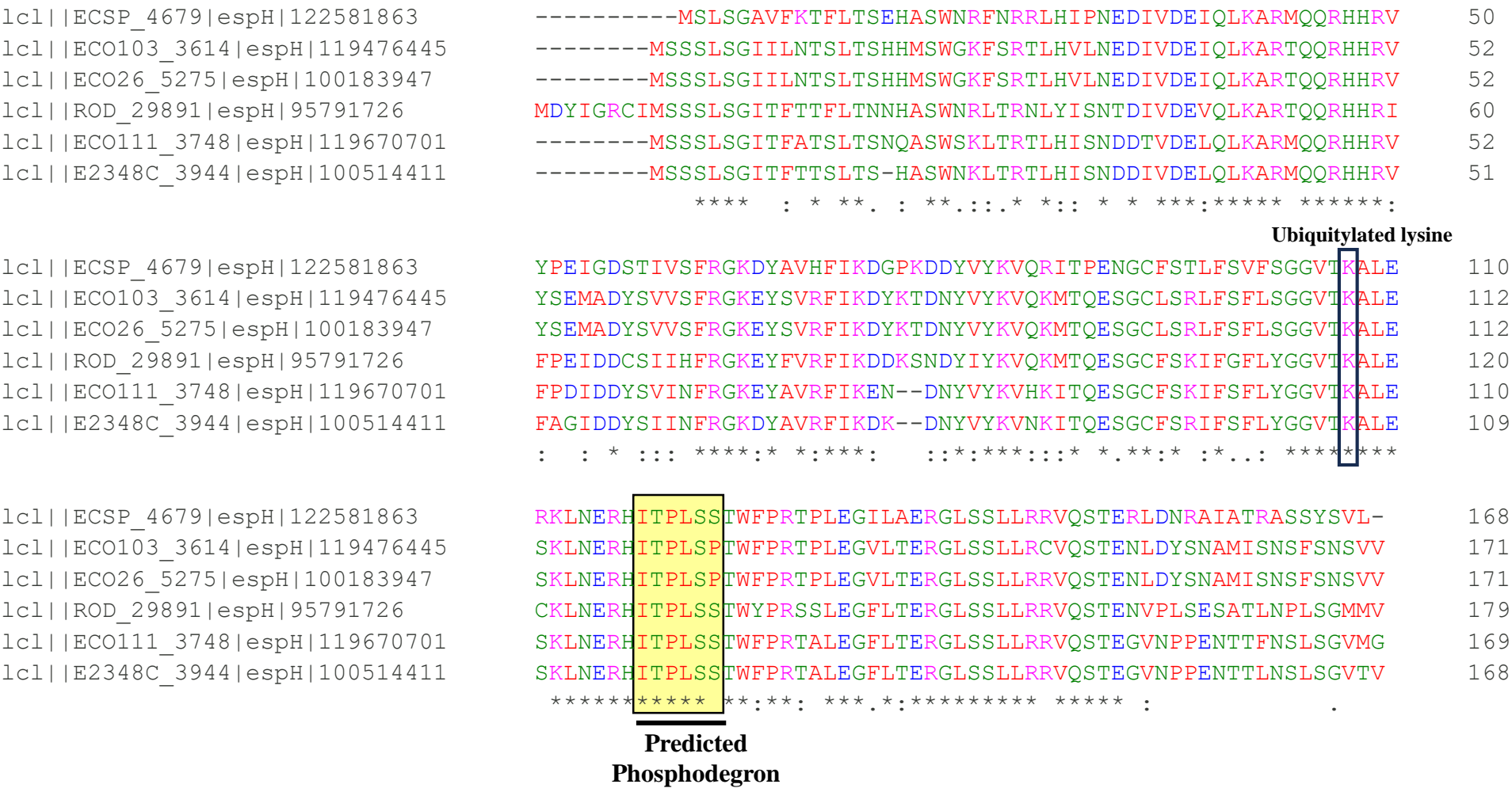

Supplement: Supplementary Material [file KGMI_A_2595775_SM3152.pdf]
