# Supplementary material for: The ubiquitin system targets translocated EspH to proteasomal degradation
Source: Gut Microbes. 2025 Dec 4;17(1):2595775. doi: 10.1080/19490976.2025.2595775 (PMC12688225; doi:10.1080/19490976.2025.2595775)
Supplement: Supplementary Material — Table S1: Cells, Bacterial Strains, Antibodies, Reagents/Kits, Materials, and Software. [file KGMI_A_2595775_SM3154.docx]

**Table S1:** **Cells, Bacterial Strains, Antibodies, Reagents/Kits, Materials and Software**

| **Reagent type (Species) or resource** | **Designation** | **Source or reference** | **Identifiers** | **Comments** |
| --- | --- | --- | --- | --- |
| **Mammalian cells** | | | | |
| Cell line  (Homo sapiens) | HeLa  cervix adenocarcinoma | ATCC – CCL-2 |  |  |
|  | CaCo-2_BBe_  colon adenocarcinoma | J. Turner; Harvard Medical School ([1](#_ENREF_1)) |  | Express similar characteristics of small intestine enterocytes ([2](#_ENREF_2)) |
| **Bacterial strains** | | | | |
| \| EPEC (E2348/69) \| Enteropathogenic *Escherichia coli* O127:H6 E2348/69 \| ([3](#_ENREF_3)) \| BA140 \| EPEC wild-type (EPEC*wt*) isolate NCBI accession no: FM180568  (Whole genome sequence) \| \| --- \| --- \| --- \| --- \| --- \| | | | | |
| EPEC (E2348/69)  Derivatives | EPEC-Δ*espH* | ([4](#_ENREF_4)) | XT111 | EPEC mutated in the *espH* gene; Kn^R^; ∆*espH::Kn* |
|  | EPEC-Δ*espH*-pEspH*_wt_* | ([5](#_ENREF_5)) | BA1104 | EPEC-Δ*espH* transformed with a pSA10 plasmid encoding EspH*_wt_* C-terminally tagged with six histidines (6XHis) and streptavidin binding peptide (SBP); ∆*espH/*pSA10-EspH*_wt_*-6XHis-SBP |
|  | EPEC-Δ*espH*-pEspH*_K106R_* | ([6](#_ENREF_6)) | BA1200 | EPEC-Δ*espH* transformed with a pSA10 plasmid encoding EspH*_K106R_* C-terminally tagged with 6XHis and SBP; ∆*espH*/pSA10-EspH*_K106R_*-6XHis-SBP |
| **Antibodies** |  |  |  |  |
| Primary  Antibodies | Anti-SBP Tag  (Mouse monoclonal) | Santa Cruz Biotechnology | Sc-101595 | 1:2000 (IB) |
|  | Anti-α-tubulin  (Mouse monoclonal) | Sigma-Aldrich | T6074 | 1:2000 (IB) |
|  | Anti-ubiquitin (mono and polyubiquitin) FK2 (Mouse monoclonal) | Cayman Chemical | 14220 | 1:1000 (IB) |
| Secondary  Antibodies | Anti-mouse IgG  (Peroxidase goat) | Jackson ImmunoResearch Laboratories | 115-035-166 | 1:10000 (IB) |
| **Reagents/Kits** |  |  |  |  |
| Media | Dulbecco's Modified Eagle Medium-high glucose (DMEM) | Sartorius (Gottingen, Germany) | 01-055-1A | Base media used for propogating cells |
|  | Phenol-red-free DMEM- high glucose | Biological Industries | 01-053-1A | Propidium Iodide (PI) assay |
|  | Luria-Bertani broth (LB) | Made in the lab |  | Bacterial growth |
| Reagents | Isopropyl β- d-1-thiogalactopyranoside (IPTG) | Promega (Madison, WI) | V3955 | Inducing EspH expression in EPEC |
|  | Ampicillin sodium salt | Sigma-Aldrich (USA) | A0166 | Antibiotics, working solution: 100 µg/ml |
|  | Kanamycin sulfate | Calbiochem (US and Canada) | 420311 | Antibiotics; working solution: 100 µg/ml |
|  | Prestained Protein Markers | BioGate (Israel) | BG-PM03 | Protein Markers for SDS-PAGE |
|  | Propidium Iodide (PI) | Sigma-Aldrich (USA) | P4170 | 5 mg/ml stock; used for PI-uptake assay |
|  | MG132, readymade solution | Sigma-Aldrich (USA) | M7449 | 10 mM in DMSO |
| Kit | CytoTox 96® Non-Radioactive Cytotoxicity Assay | Promega, Wisconsin, USA | G1780 | Used for LDH release assay |
| Inhibitors | Protease and phosphatase inhibitors (PPI) | Sigma-Aldrich (USA) | PPC1010 | 1:100; PPI: buffer (ratio) |
| Protein quantification | Bradford Reagent | Sigma-Aldrich (USA) | B6916 | For 1-1.4 µg/ml protein |
| Beads | Streptavidin (StAv) agarose beads | Sigma-Aldrich (USA) | S1638 | Used to precipitate EspH-6xHis-SBP |
| **Materials** |  |  |  |  |
| Tissue culture plates | Nunc dish 100X15 (10-cm plate) | Thermo Fisher Scientific (Waltham, Massachusetts, USA) | 150350 |  |
|  | Nunc dish 150X20 (15-cm plate) | Thermo Fisher Scientific (Waltham, Massachusetts, USA) | 168381 |  |
|  | Nunc multi-dish (6-well plate) | Thermo Fisher Scientific (Waltham, Massachusetts, USA) | 140685 |  |
|  | Flat bottom 96-well plate | Greiner (Kremsmunster, Austria) | 655090 |  |
| **Software and algorithms** | | | | |
| Software | GraphPad Prism v. 8.4.3 | GraphPad Software (Massachusetts, USA) |  | Graph preparation and statistical analysis |
|  |  |  |  |  |

**References**

1. Shen L, Black ED, Witkowski ED, Lencer WI, Guerriero V, Schneeberger EE, Turner JR. 2006. Myosin light chain phosphorylation regulates barrier function by remodeling tight junction structure. J Cell Sci 119:2095-106.

2. Peterson MD, Mooseker MS. 1992. Characterization of the enterocyte-like brush border cytoskeleton of the C2BBe clones of the human intestinal cell line, Caco-2. J Cell Sci 102 ( Pt 3):581-600.

3. Levine MM, Bergquist EJ, Nalin DR, Waterman DH, Hornick RB, Young CR, Sotman S. 1978. Escherichia coli strains that cause diarrhoea but do not produce heat-labile or heat-stable enterotoxins and are non-invasive. Lancet 1:1119-22.

4. Tu X, Nisan I, Yona C, Hanski E, Rosenshine I. 2003. EspH, a new cytoskeleton-modulating effector of enterohaemorrhagic and enteropathogenic Escherichia coli. Mol Microbiol 47:595-606.

5. Ramachandran RP, Vences-Catalan F, Wiseman D, Zlotkin-Rivkin E, Shteyer E, Melamed-Book N, Rosenshine I, Levy S, Aroeti B. 2018. EspH Suppresses Erk by Spatial Segregation from CD81 Tetraspanin Microdomains. Infect Immun 86.

6. Nandi I, Ramachandran RP, Shalev DE, Schneidman-Duhovny D, Shtuhin-Rahav R, Melamed-Book N, Zlotkin-Rivkin E, Rouvinski A, Rosenshine I, Aroeti B. 2024. EspH utilizes phosphoinositide and Rab binding domains to interact with plasma membrane infection sites and Rab GTPases. Gut Microbes 16:2400575.
